# Interactions at the point of firearm purchase and subsequent use of locking devices

**DOI:** 10.1186/s40621-023-00421-0

**Published:** 2023-03-01

**Authors:** Shelby L. Bandel, Allison E. Bond, Michael D. Anestis

**Affiliations:** 1 Rutgers School of Public Health, New Jersey Gun Violence Research Center, 683 Hoes Lane West Piscataway, Piscataway, NJ 08854 USA; 2 Department of Psychology, The State University of New Jersey, 683 Hoes Lane West Piscataway, Rutgers, NJ 08850 USA

**Keywords:** Firearms, Safe firearm storage, Suicide prevention

## Abstract

**Background:**

Firearms account for over 40,000 deaths in the USA each year in addition to thousands of nonfatal injuries. One notable prevention strategy for firearm-related fatalities and nonfatal injuries is safe storage. Safe storage of firearms emphasizes using storage mechanisms that limit ready access of firearms to unauthorized users. Cable locks are one safe storage option that is easy to access and typically free, as they are included in many firearms sales. The present study examined the extent to which firearms retailers notifying purchasers at the point of sale about the included cable locks was associated with subsequent locking device use in two large samples and three subsamples. Exploratory analyses then examined demographic factors associated with frequency of seller notification of locks.

**Methods:**

Sample 1 included 1203 firearm owners and sample 2 included 1556 firearm owners. Subsamples were drawn from sample 2 to examine if there were differences by state. The three subsamples included firearm owners from Minnesota (*n* = 515), Mississippi (535), and New Jersey (506). Logistic regressions were used to examine the association between frequency of sellers notifying buyers of locks and subsequent locking device use. Linear regressions were used to examine what demographic factors were associated with greater frequency of seller notification of locking devices.

**Results:**

Results indicated a general trend such that more frequent notification of cable locks at the point of purchase was associated with greater likelihood of using locking devices to secure firearms. At the subsample level, these findings were most consistent for Mississippi relative to Minnesota and New Jersey. Exploratory analyses generally indicated those who were younger and those living in more densely populated areas were more likely to be notified about cable locks at the point of purchase.

**Conclusions:**

These findings suggest that interactions about cable locks at the point of firearm purchase has an impact on firearm storage behaviors. Such results indicate that encouraging firearm retailers to have these discussions with those purchasing firearms might be an important strategy for preventing firearm-related fatalities and nonfatal injuries.

## Background

Firearms account for nearly 40,000 deaths in the United States (USA) each year, the majority of which occur by suicide (Centers for Disease Control and Prevention 2022). Firearms are utilized in roughly 50% of suicide deaths in the USA each year despite being used in fewer than 5% of suicide attempts (Conner et al. 2007). This discrepancy is the result of firearms having a 90% case fatality rate when used in suicide attempts (Spicer and Miller 2000). Additionally, although a precise count is difficult to formulate, it is estimated that there are over 85,000 nonfatal firearm-related injuries in the USA each year (Kaufman et al. 2020 Dec 7) indicating that firearms not only account for many deaths each year but are also responsible for numerous nonfatal injuries.

Given the prevalence of firearm fatalities and firearm-related injuries in the USA each year, it is evident that solutions to combat gun violence are necessary. One potential avenue to preventing firearm-related injuries and death is safe firearm storage. Safe storage of firearms emphasizes using storage mechanisms that limit ready access of firearms to unauthorized users. Safe storage mechanisms include storing firearms unloaded and separate from ammunition, in a lock box/safe, or with cable locks/trigger locks in place. Some of these storage methods (e.g., safe, lock box) require firearm owners to purchase devices separately from their firearm, whereas others (e.g., cable/trigger locks) are made readily available upon purchasing a firearm. This availability is due to several pieces of legislation over the past three decades. For example, beginning in 1998, federal firearms dealers have been required to certify that gun storage or safety devices are available anywhere firearms are sold, per the Omnibus Consolidation and Emergency Supplemental Appropriations Act. In 2005, the Child Safety Lock Act was passed, requiring that locking devices (e.g., cable locks) be included with nearly all handgun purchases in the USA. Further, although the Child Safety Lock Act only specifies that locking devices be included with handgun sales, firearms manufacturers have frequently included locking devices with multiple types of new firearms when are shipped from the factory. Most commonly, the devices provided are cable locks. The ready availability of cable locks suggests that they might pose an important avenue for safe storage and thus may be an important area to target as a way to reduce firearm-related injuries and fatalities.

Despite the importance of safe storage, we know very little about how individuals select storage methods for their firearms. Prior research has demonstrated that there are factors associated with safe firearm storage in general, finding that having children in the home, only owning handguns, and being exposed to firearm safety courses are associated with greater likelihood of safe storage (Crifasi et al. 2016). With regard to cable lock use specifically, even less is known; however, one prior study found that participants who were provided cable locks within the context of a randomized controlled trial of lethal means counseling were more likely to store their firearms using cable locks at subsequent follow-up sessions (Anestis et al. 2021). These findings suggest that providing individuals cable locks may aid in safe firearm storage. Notably, results from a different study conducted among law enforcement officers suggests simply supplying cable locks may not be enough. Specifically, researchers found that, among law enforcement officers who were given free cable locks, nearly two thirds reported not using these locks to secure their firearms (Coyne-Beasley and Johnson 2001). Similarly, in a large representative national sample, Crifasi and colleagues (Crifasi et al. 2016). found that only 24% of firearm owners used trigger/other locks as the sole form of firearm safety for all firearms in their home whereas 56% did not use trigger/other locks for any firearms in their home. It is evident that simply supplying individuals with a cable lock does not ensure that individual will utilize it and more research is needed to better understand how to promote consistent use of locking devices supplied at the point of purchase.

While little is known about how to facilitate cable lock use in the USA, there continues to be a focus on increasing the distribution of cable locks to increase safe storage. For example, the Biden administration has taken several steps to increase cable lock use as part of a broader safe storage effort. This has included an initiative though the Department of Veterans Affairs (VA) to launch a public service campaign reinforcing the idea that firearm locks can save lives (The White House 2021). Further, a recently finalized rule by The Bureau of Alcohol, Tobacco, Firearms and Explosives (ATF) requires firearm dealers to ensure compatible firearm storage/safety devices are available for purchase at the place of firearm sales (The White House 2021). ATF is also issuing best practice guidelines to dealers that reinforce the steps dealers are required to take in addition to steps they are encouraged to take as a way to keep their communities and customers safe (The White House 2021). These guidelines also include materials that are to be distributed to customers that explain firearm owners’ legal obligations and steps they can take to facilitate safe storage (The White House 2021). Additionally, there have been several initiatives within the Department of Defense (DoD) outside of the current administration’s efforts to increase cable lock accessibility to military service members. Specifically, the National Defense Authorization Act allowed commanders and clinicians to encourage safe storage. Further, the Air Force recently distributed 150,000 cable locks to every installation in the USA for dissemination to service members in conjunction with a safe storage conversation to aid in proper weapon use and storage (Guns and America 2020). While the distribution of cable locks alone may be an important step in firearm injury prevention, little is known about who these cable locks have reached and whether these efforts have resulted in cable lock use or prevented deaths.

Given that cable locks are the most widely available avenue for safe storage and the current initiatives to highlight these as a storage option, the present study sought to better understand how interactions at the point of purchase might impact cable lock use. First, we examined if there was an association between the frequency with which firearm owners reported that firearm dealers notified them of the cable locks included in their firearm purchase and the actual use of cable locks to secure firearms in two large samples of firearm owners. The first sample was comprised of participants from the entire US and the second was comprised of individuals from Minnesota, Mississippi, and New Jersey. Knowing if interactions at the point of purchase are related to subsequent storage behavior could provide valuable information for determining if these sorts of interactions need to be encouraged more often as a way to facilitate safe storage. We then examined the three states (Minnesota, Mississippi, and New Jersey) from the second sample separately to determine if different results arise when states have different political leanings, firearm ownership rates, and firearm ownership laws. Such findings allow us to better understand if the influence of notifications at the point of purchase is similarly effective with such differences. Finally, we conducted exploratory analyses to determine if purchaser demographic characteristics (i.e., race, age, gender, rurality) were associated with sellers’ frequency of notifying buyers of cable locks. These findings would allow us to better understand who is more/less likely to get this information at the point of purchase and thus may identify specific groups that retailers need to be encouraged to engage in conversations regarding cable locks. Although preliminary and largely descriptive, these findings offer potential value in that they may provide information about a firearm safety strategy that could be easily disseminated to many who purchase firearms.

## Methods

### Procedures

The present study utilized two separate samples. In the first sample, a subset of firearm owning (*n* = 1,203) participants from a large online survey (*N* = 3,500) seeking to assess firearm perceptions within the USA was used. This sample was recruited with the help of Qualtrics Panels, who utilized quota sampling to match participants to the 2010 census data on age, race, sex, income, and education level. Additionally, in the second sample, a subset of firearm owning (*n* = 1,556) participants from a large online survey seeking to understand firearm perceptions (*N* = 6,404) was used. Again, Qualtrics Panels used quota sampling to match participants to statewide 2010 Census demographics; however, participants only came from Minnesota (*n* = 515), Mississippi (*n* = 535), and New Jersey (*n* = 506). Within Minnesota, participants were oversampled from ZIP codes within the Twin Cities. Precise participation rates are difficult to calculate due to the nature of quota sampling; however, Qualtrics estimates a participation rate of 57% for the national sample and 59% for the three state sample.

Both studies received necessary Institutional Review Board approval and consent was obtained from all participants prior to starting the survey. Participants were compensated at the price they agreed upon with Qualtrics Panels. Quality assurance items were included in both samples (e.g., have you ever used a computer?).

### Measures

A majority of measures were consistent across samples. Demographic information was assessed using measures created by the research team and these measures have been used in multiple other studies.

Questions assessing notification of cable locks at time of purchase differed in the two samples and were developed by the research team. Both samples asked participants to retrospectively report on the frequency they recall having been notified of locking devices included in firearms purchases at the point of sale. Specifically, in the first sample, participants were asked, “Were you explicitly told about the locking device included in the firearm purchase by the seller?” Response options included “for none of my firearm purchases,” “for a few of my firearm purchases,” “for some of my firearm purchases,” “for most of my firearm purchases,” and “for all of my firearm purchases.” In the second sample participants were asked, “During what percentage of your firearm purchases were you explicitly told about the locking device included in the firearm purchase by seller?” Response options were the same as in the first sample with one addition, “for many of my firearm purchases” and one alteration, "for most of my firearm purchases" became "for almost all of my firearm purchases."

In both samples, current firearm storage habits were assessed with the following question, “Which of the following storage procedures do you use for the firearms currently located in or around your home? (Select all that are used).” Responses included, “gun safe,” “gun cabinet,” “locking device (e.g., trigger lock, cable lock),” “hard case (e.g., pelican case), hide in closet or drawer, unloaded,” “hide in closet or drawer, loaded,” and “other safety procedures.”

### Data analytic section

Logistic regressions controlling for number of firearms owned were used to examine the frequency of seller notification of cable locks and utilization of cable locks. We controlled for the number of firearms owned to ensure that those who only owned one firearm were not impacting the results more than those who owned multiple firearms because those who owned one firearm would only have response options at the extreme end of the scale whereas those with multiple firearms could have responses across the entire scale. For all logistic regressions, the reference group in the cable lock notification comparisons was “For none of my firearms purchases.” Linear regressions were utilized for the exploratory analyses. Notably, for the national and three state samples all racial groups and ethnicity were examined in the exploratory analyses; however, due to limited sample sizes across the subsamples (Minnesota, Mississippi, and New Jersey) race was examined as white/non-white and ethnicity was excluded.

## Results

Sample demographic characteristics are presented in Tables 1.

**Table 1:** Demographic characteristics for all samples

	National representative sample (*N* = 1,203) *N* (%)	Three state sample (*N* = 1,556) N (%)	Minnesota (*n* = 515) *N* (%)	Mississippi (*n* = 535) *N* (%)	New jersey (*n* = 506) *N* (%)
Age					
M (SD)	45.04 (16.43)	45.53 (17.3)	48.07 (17.41)	41.75 (16.54)	46.33 (16.94)
Range	18–91	18–85	18–83	18–84	18–85
Sex					
Male	767 (63.8%)	905 (58.2%)	323 (62.7%)	218 (40.7%)	364 (71.9%)
Female	436 (36.2%)	651 (41.8%)	192 (37.3%)	317 (59.3%)	142 (28.1%)
Race					
White	890 (73.9%)	1223 (78.6%)	462 (89.7%)	348 (65%)	413 (81.6%)
Black	162 (13.5%)	256 (16.5%)	27 (5.2%)	168 (31.4%)	61 (12.1%)
Asian	70 (5.8%)	49 (3.1%)	17 (3.3%)	9 (1.7%)	23 (4.5%)
American Indian/ Alaska Native	56 (4.7%)	38 (2.4%)	10 (1.9%)	21 (3.9%)	7 (1.4%)
Native Hawaiian/ Pacific Islander	14 (1.2%)	13 (0.8%)	7 (1.4%)	4 (0.7%)	2 (0.4%)
Other	38 (3.2%)	18 (1.2%)	5 (1%)	5 (0.9%)	8 (1.6%)
Ethnicity					
Hispanic/Latino	147 (12.2%)	97 (6.3%)	15 (2.9%)	26 (4.9%)	56 (11.1%)
Non-Hispanic/Latino	1057 (87.8%)	1453 (93.7%)	498 (97.1%)	507 (95.1%)	448 (88.9%)
Firearms Owned					
M (SD)	3.9 (16.4)	4.8 (5.0)	5.3 (5.2)	4.9 (5.0)	4.3 (4.9)
% using locking devices on 1 + firearm	30.3%	32%	31.1%	29.7%	35.4%

### Primary analyses

Logistic regression results for all primary analyses are presented in Tables 2 and 3.

**Table 2:** Logistic regressions predicting cable/trigger lock use

	OR	Wald	95% CI
National representative sample			
Number of firearms	1.01	.47	.98, 1.04
For a few of my firearm purchases	2.38	3.83	.99, 5.65
For some of my firearm purchases	2.79	6.69	1.28, 6.06
For most of my firearm purchases	3.18	8.54	1.46, 6.92
For all of my firearm purchases	4.27	16.04	2.10, 8.70
	*χ*^2^	df	*p*
Omnibus test	22.82	5	< .01
Three State Sample			
Number of Firearms	.98	3.58	.95, 1.00
For a few of my firearm purchases	1.57	2.34	.88, 2.80
For some of my firearm purchases	2.19	9.57	1.33, 3.59
For many of my firearm purchases	2.06	7.75	1.24, 3.43
For almost all of my firearm purchases	2.93	14.21	1.68, 5.13
For all of my firearm purchases	2.86	19.26	1.79, 4.58
	χ^2^	df	*p*
Omnibus test	31.72	6	< .01

For both the national representative sample and three state sample the reference group for frequency of retailers noting cable locks at the point of purchase is “For none of my firearms”

**Table 3:** Logistic regressions predicting cable lock use in Minnesota, Mississippi, and New Jersey

	Minnesota			Mississippi			New Jersey		
	OR	Wald	95% CI	OR	Wald	95% CI	OR	Wald	95% CI
Number of Firearms	.98	.75	.94, 1.03	.95	6.45	.91, .99	1.00	.03	.96, 1.04
For a few of my firearm purchases	.88	.07	.327, 2.35	2.69	4.00	1.02, 7.06	1.49	.46	.48, 4.64
For some of my firearm purchases	2.33	3.80	.99, 5.47	1.97	2.31	.82, 4.69	2.17	2.50	.83, 5.68
For many of my firearm purchases	1.84	1.87	.77, 4.43	3.06	6.19	1.27, 7.38	1.57	.81	.59, 4.18
For almost all of my firearm purchases	2.99	5.35	1.18, 7.56	3.66	7.07	1.41, 9.51	2.15	1.88	.72, 6.42
For all of my firearm purchases	2.06	3.46	.96, 4.40	4.04	11.28	1.79, 9.13	2.59	3.97	1.02, 6.60
	*χ*^2^	df	*p*	*χ*^2^	df	*p*	*χ*^2^	df	*p*
Omnibus test	11.82	6	.07	24.66	6	< .01	6.66	6	.35

For all three states the reference group for frequency of retailers noting cable locks at the point of purchase is “For none of my firearms”

#### Sample 1

After controlling for the total number of firearms individuals had, results indicated a general trend such that greater frequency of sellers notifying firearm owners of cable locks at the point of purchase was associated with increased likelihood of current locking device use. Specifically, those who reported being told about cable locks “For some of my firearm purchases” (OR = 2.79, *p* = 0.01, 95% CI = 1.28, 6.06), “For most of my firearm purchases” (OR = 3.18, *p* < 0.01, 95% CI = 1.46, 6.92), and “For all of my firearm purchases” (OR = 4.27, *p* < 0.001, 95% CI = 2.10, 8.70) were significantly more likely to use locking devices relative to those who were told about “For none of my firearm purchases.”

#### Sample 2

Similar to sample 1, after controlling for the total number of firearms individuals had, results indicated a general trend such that greater frequency of sellers notifying firearm owners of cable locks at the point of purchase was associated with increased likelihood of current locking device use. Specifically, those who were told about cable locks “For some of my firearm purchases” (OR = 2.19, *p* < 0.01, 95% CI = 1.33, 3.59), “For many of my firearm purchases” (OR = 2.06, *p* < 0.01, 95% CI = 1.24, 3.43), “For almost all of my firearm purchases” (OR = 2.93, *p* < 0.001, 95% CI = 1.68, 5.13), and “For all of my firearm purchases” (OR = 2.86, *p* < 0.001, 95% CI = 1.79, 4.58) were significantly more likely to use locking devices relative to those who were told about cable locks “For none of my firearm purchases.”

#### Minnesota

After controlling for the total number of firearms individuals had, results indicated that those who were told about cable locks “For almost all of my firearm purchases” were significantly more likely to use locking devices relative to those who were told about cable locks “For none of my firearm purchases” (OR = 2.99, *p* = 0.02, 95% CI = 1.18, 7.56). All other comparisons were nonsignificant.

#### Mississippi

After controlling for the total number of firearms individuals had, results indicated a general trend such that greater frequency of sellers notifying firearm owners of cable locks at the point of purchase was associated with increased likelihood of locking device use. Specifically, those who were told about cable locks “For a few of my firearm purchases” (OR = 2.69, *p* = 0.04, 95% CI = 1.02, 7.06), “For many of my firearm purchases” (OR = 3.06, *p* < 0.01, 95% CI = 1.41, 9.51),“For almost all of my firearm purchases” (OR = 3.66, *p* < 0.01, 95% CI = 1.41, 9.51), and “For all of my firearm purchases” (OR = 4.04, *p* < 0.001, 95% CI = 1.79, 9.13) were significantly more likely to use locking devices relative to those who were told about cable locks “For none of my firearm purchases.”

#### New jersey

After controlling for the total number of firearms individuals had, results indicated that those who were told about cable locks “For all of my firearm purchases” were significantly more likely to use locking devices relative to those who were told about cable locks “For none of my firearm purchases” (OR = 2.59, *p* = 0.046, 95% CI = 1.02, 6.60). All other comparisons were nonsignificant.

### Exploratory analyses

Linear regression analyses for all exploratory analyses examining the association between demographic factors (age, sex, race, and rurality) and the frequency which individuals reported being notified of cable locks are presented in Tables 4.

**Table 4:** Linear regressions of demographic characteristics predicting frequency of seller notification of cable locks at the point of purchase for the national representative sample and the Minnesota subsample

	National representative sample					Minnesota			
	*B*	*p*	95% CI	F^2^	*B*	*p*	95% CI	F^2^	
Age	− .13	< .01	− .02,-.00	.01	Age	− .16	< .01	− .03, -.01	.02
Sex (Female)	− .04	.28	− .31, .09	.00	Sex (Female)	.01	.81	− .37, .47	.00
Race					Race				
Black	− .03	.45	− .38, .17	.00	White	− .04	.53	− .75, .39	.00
Asian	− .05	.14	− .64, .09	.00	Rurality				
American Indian/ Alaska Native	− .03	.41	− .65, .27	.00	Metropolitan Rural	.22	< .01	.44, 1.51	.04
Native Hawaiian/ Pacific Islander	− .04	.23	− 1.20, .29	.00	Urban	.14	.03	.05, .98	.02
Other	− .01	.71	− .69, .47	.00					
Ethnicity									
Hispanic/Latino	.03	.50	− .19, .40	.00					
Rurality									
Metropolitan Rural	.07	.09	− .03, .43	.00					
Urban	.13	.00	.12, .57	.01					

#### Sample 1

Results indicated that the overall model was significant (*F* = 2.48, *p* < 0.01). Investigation of specific variables indicated that individuals who reported more frequent notification of cable locks at the point of purchase were younger (*ß* = − 0.13, *p* < 0.01, *F*^2^ = 0.01) and more likely to live in an area classified as urban relative to rural (*ß* = 0.13, *p* < 0.01, *F*^2^ = 0.01).

#### Sample 2

Results indicated that the overall regression model was not significant (*F* = 1.30, *p* = 0.22) as such, specific variables were not examined.

#### Minnesota

Results indicated that the overall model was significant (*F* = 5.06, *p* < 0.01). Investigation of specific variables indicated that individuals who reported more frequent notification of cable locks at the point of sale were younger (*ß* = − 0.16, *p* < 0.01, *F*^2^ = 0.02), more likely to live in an area classified as metropolitan-rural relative to rural (*ß* = 0.22, *p* < 0.01, *F*^2^ = 0.04), and were more likely to live in an area classified as urban relative to rural (*ß* = 0.14, *p* = 0.03, *F*^2^ = 0.02).

#### Mississippi

Results indicated that the overall regression model was not significant (*F* = 1.50, *p* = 0.19) as such, specific variables were not examined.

#### New jersey

Results indicated that the overall regression model was not significant (*F* = 1.39, *p* = 0.23) as such, specific variables were not examined.

## Discussion

Findings from the present study indicated a general trend such that those who reported recalling more frequent notification from firearms retailers at the point of purchase about the included cable lock included with firearms sales had a greater likelihood of storing firearms using cable/trigger locks. Specifically, the more often individuals reported they were informed about cable locks included in firearms purchases at the point of sale, the more likely they were to use cable/trigger locks as a way to secure their firearms. This general trend may suggest that consistent notification of cable locks is particularly important in facilitating safe firearm storage. As such, these findings advocate for firearms retailers to note the cable locks included in firearms purchases every time an individual buys a firearm. This approach is particularly notable because it requires relatively minimal effort, especially in comparison to other means safety approaches (e.g., lethal means counseling) and results in meaningful increases in likelihood of locking device use. Notably, we do not know what these conversations looked like (e.g., the length of time these conversations occurred, what the content of the conversations was, etc.) and such factors might have an impact on individuals’ decision to utilize these mechanisms to secure their firearms.

Notably, given that this data was retrospective self-report, it is impossible to determine if these behaviors arose following the interactions with firearm retailers. Specifically, it may be that the results are impacted by recall bias such that those who use locking devises are more likely to recall conversations at the point of purchase. It is possible that these conversations are occurring during more firearms purchases than the results reflect and those who do not use locking devices simply do not recall these conversations whereas those who do use locking devices more readily remember the interaction. Nevertheless, given the consistency of the findings across multiple samples, it appears as though interactions during firearms sales may have some impact on locking device use and more longitudinal research is needed to determine the directionality of this interaction.

Prior research on who firearm owners find credible to discuss safe storage has found that firearms sellers and manufactures typically rank relatively low in their perceived credibility, both for general firearm safety information and firearm suicide prevention (Crifasi et al. 2016; Bond et al. 2022). The fact that the present study found that firearms retailers may have an impact on firearm storage behaviors despite this potential limited perceived credibility is particularly notable. It may suggest that even those who are perceived to be less credible can still have a notable impact on firearm owners’ storage behaviors if the conversation occurs in an appropriate context. As such, it may be concluded that we should not dismiss the potential impact of those ranked lower in credibility, as they may still influence firearm safety behaviors. Alternatively, such findings may suggest that there is a disconnect between who people rank as credible and who they actually perceive to be credible. For example, while completing surveys, firearm owners may think that firearm retailers are not credible; however, when put in positions where they have interactions with firearms retailers, the credibility of these sources may be greater than what was perceived in outside settings (e.g., research studies).

These findings suggest that it is important for there to be some sort of interaction about cable locks in distribution efforts. Therefore, simple efforts to distribute cable locks without conversations may prove to be ineffective in increasing safe firearm storage. Consistent with this notion, in a recent clinical trial demonstrating that those provided cable locks were more likely to store firearms safely, these individuals were not simply handed the locks in the absence of a conversation (Anestis et al. 2021). Instead, individuals were engaged in a discussion of how many locks they wanted as well as instructions on how to use them if individuals stated they were unfamiliar with how to properly affix them to firearms (Anestis et al. 2021). Unfortunately, many prior cable lock distribution efforts within the VA have provided cable locks in the absence of conversations (Nalpathanchil 2013),instead opting to leave cable locks at tables for individuals to pick up as they see fit. However, the recent initiative in the Air Force to distribute cable locks does include an educational conversation component (Guns and America 2020). Findings from the present study indicate that the shift to distributing cable locks in conjunction with such conversations is likely an important step to increasing safe firearm storage.

Prior to the present study, research had begun to examine how more in-depth discussions can have an impact on storage of potential suicide methods. For example, research in emergency departments has found that following lethal means counseling parents were more likely to store both firearms and medications securely relative to the control (Miller et al. 2020). Further, the comfort of medical professionals in conducting lethal means counseling can be improved with training, suggesting it is possible to implement more widespread dissemination of such conversations (Salhi et al. 2021). Similarly, a recent clinical trial conducted among service members demonstrated that following one brief (15–20 min) lethal means counseling session focused on firearm safety, service members were more likely to store firearms safely at subsequent follow-ups relative to those who did not receive the lethal means counseling (Anestis et al. 2021). While these prior studies shed light on the potential impact of more in depth firearm storage conversations, findings from the present study suggest that even shorter conversations – at least within the context of firearm sales, where in group perceptions may be high – could also yield meaningful results in firearm storage behavior.

Of note were state differences in the association between frequency of seller notification and lock use. Specifically, the state that showed the greatest number of significant associations and largest impact was Mississippi. Mississippi represented the state with the highest firearm ownership rate in addition to being the most politically conservative state among the three states (Pew Research Center 2022; RAND Corporation 2022) Finding that seller notification was associated with increased cable lock use among a state like Mississippi is important as those who are more conservative are typically considered to be a harder to reach group. If sellers’ more frequent notification of cable locks at the point of purchase has such an impact in Mississippi, these interactions may have particular weight in highly conservative states with high rates of ownership. Arguably, these are some of the most important states we need to be reaching with safe firearm storage efforts as they are often states with higher rates of suicide and unintentional firearm-related deaths (Centers for Disease Control and Prevention 2022).

Exploratory analyses from the present study suggest that there might be demographic factors associated with how frequently individuals are informed about included cable locks at the point of firearm purchase. Specifically, younger age was associated with increased notification of locking devices included in firearm sales. This may be due to a couple of factors. First, it may be that retailers see younger firearm owners as more novice and thus may be more likely to tell them about the locks based on an assumption that younger people do not know about this storage mechanism. Second, it may be that the changes in laws surrounding cable locks in addition to shifts in firearm manufactures practice of including locking devices over time is having an impact on these results. Specifically, legislation requiring cable locks inclusion in all handgun sales was passed in 2005 around which time firearms manufacturers began including cable locks with all firearms from the factory. Therefore, it may be that older firearm owners purchased firearms before such actions took place and thus have less frequently been notified at the point of purchase relative to younger firearm owners. It is important for future research to examine this association to determine if this finding is a result of changes in practice over time or if there are differences in how retailers view firearm purchasers and thus interact with them differently as it related to cable lock discussions.

Additionally, findings indicated a difference in the frequency of notification based on an individual’s rurality. Specifically, in the nationally representative sample (Sample 1), those living in an urban area reported being notified at an increased frequency relative to those who lived in a rural area. Such finding indicates that those in more populated areas are told about cable locks at increased frequency. This may suggest that there is a perception that those who live in rural areas possess more knowledge about firearms and thus firearms retailers do not feel the need to inform them about the cable locks. Alternatively, those living in more populated areas may be perceived as more novice to firearm retailers and thus, they are more likely to inform them of the cable locks. An additional possibility is that firearm retailers in more rural areas see less value in locking devices and are thus less motivated to discuss them with their customers.

While the three state sample overall was nonsignificant, when examining the three states (Minnesota, Mississippi, and New Jersey) independently, results indicated that there were several demographic factors associated with frequency of notification in Minnesota. Specifically, within Minnesota results indicated that those who reported more frequent cable lock notification were younger and more likely to live in a metropolitan-rural or urban area (relative to a rural area). It is noteworthy that these results were only evident in the Minnesota subsample. These results suggest that there is more variation in cable lock notification based on demographic characteristics in Minnesota relative to Mississippi and New Jersey. Notably, more than half of the Minnesota sample lived in Twin Cities ZIP codes, thereby providing a large urban sample within a state marked by many rural communities. As such, the geographic distribution of the participants within this particular state may have directly impacted the results relative to the other two states.

Limitations from the present study are worth noting. First, we asked for firearm owners to retrospectively report on the frequency at which they were notified about cable locks. Such retrospective report can be impacted by hindsight bias and may not be entirely accurate. Second, our question assessing cable lock use simultaneously asked about trigger lock use as the two storage practices were combined into one response option. As such, our primary results are also accounting for those using trigger locks while our true interest is the use of cable locks. Future research should aim to replicate these results while specifically asking about cable lock use. Third, we did not assess which firearm individuals use cable locks for; specifically, we do not know if individuals are using the cable lock on a firearm which they were told about the cable lock at the point of purchase or if they are using cable locks for other firearms. Forth, as previously mentioned, we do not have information about the content and length of the conversations about the included cable locks at the point of purchase. As a result, it is difficult for us to provide firearms retailers specific information about how long these conversations need to be or what the nature of these conversations should be to facilitate behavior change. Additionally, our use of quota sampling rather than probability-based sampling decreased our ability to speak to the representativeness of the sample. Further, given that data was collected online, those living in more rural areas may have lacked internet access necessary for participation. Moreover, those with internet access living in rural areas may be more hesitant to participate in online surveys. As such, the generalizability of our finding to those living in such areas is limited. Finally, we do not know the years which firearm owners in the present sample purchased their firearms and as such they may have purchased firearms before legislative and firearms manufacture practices changed to include cable locks in sales. Future research should seek to replicate these findings while only assessing for firearm purchases following these changes.

## Conclusions

Overall, findings from the present study suggest that, by notifying those purchasing firearms that cable locks are included with the firearm sale, firearms retailers may have a positive impact on safe firearm storage. These findings have notable implications, as more widespread adoption of such practices may be useful in helping to prevent firearm suicide and unintentional firearm deaths. Additionally, there may be demographic factors that impact how likely retailers are to inform individuals about the included cable locks and as such, encouraging individuals to mention these with all sales, regardless of purchaser demographic characteristics may be important to ensure all individuals are receiving safe storage information. The present study offers hope for increasing safe firearm storage by encouraging an interaction that requires relatively minimal effort and could be implemented broadly.

## Data Availability

The datasets used and/or analyzed during the current study are available from the corresponding author on reasonable request.

## Abbreviations

USA: United States
VA: Department of veterans affairs
ATF: The Bureau of alcohol, tobacco, firearms and explosives
DoD: Department of defense
